# The Therapeutic Effect of Catechin on Nephrolithiasis Induced by Co-Exposure to Melamine and Cyanuric Acid in Sprague–Dawley Rats

**DOI:** 10.3390/toxics11090799

**Published:** 2023-09-21

**Authors:** Hangwei Wang, Zhanren Liu, Shaojie Liu, Ruoru Yang, Yifei Wang, Yiying Gu, Min Wu, Ruihua Dong, Bo Chen

**Affiliations:** Key Lab of Public Health Safety of the Ministry of Education, School of Public Health, Fudan University, Shanghai 200032, China; wanghw199912@163.com (H.W.); 23111020068@m.fudan.edu.cn (Z.L.); liushaojie@fudan.edu.cn (S.L.); 20211020125@fudan.edu.cn (R.Y.); 22111020064@m.fudan.edu.cn (Y.W.); 21211020071@m.fudan.edu.cn (Y.G.); wumin@shmu.edu.cn (M.W.); ruihua_dong@fudan.edu.cn (R.D.)

**Keywords:** melamine, cyanuric acid, nephrolithiasis, catechin

## Abstract

This study aimed to assess the therapeutic efficacy of catechin against experimentally induced kidney stones resulting from co-exposure to melamine (MEL) and cyanuric acid (CYA) in male Sprague–Dawley rats. To induce nephrolithiasis, a combination of MEL and CYA (1:1 ratio, each at a dose of 31.5 mg/kg bw/day) was administered to the rats for 28 consecutive days. After nephrolithiasis was successfully induced, the rats were randomly divided into two groups: a treatment group and a sham group. The treatment group was given a daily oral dose of 50 mg/kg of catechin for 28 days, while the sham group received no intervention. Urine and blood samples were collected throughout the treatment period, and kidney samples were taken on day 28. Our findings demonstrated that treatment with catechin significantly reduced crystal deposition and pathological damage in the rats from nephrolithiasis. Additionally, renal injury markers were significantly decreased in the treatment group compared to the sham group. These findings suggest that catechin has potential therapeutic benefits in treating nephrolithiasis induced by co-exposure to MEL and CYA.

## 1. Introduction

Urolithiasis, a prevalent urologic disorder, is caused by a complex interplay of genetic and environmental factors, with its mechanism yet to be fully understood [1]. There has been a progressive increase in the global prevalence of urolithiasis, with rates ranging from 5–10% in Europe, approximately 4% in South America, and varying between 1 and 19% across Asia [2,3,4]. In mainland China, the prevalence of kidney stones (or nephrolithiasis as a sub-group of urolithiasis) in the years 1991–2000, 2001–2010, and 2011–2016 was 5.95%, 8.86%, and 10.63%, respectively [5], which indicates a dramatically increasing trend. The most common types of urolithiasis are composed of calcium oxalate, uric acid, struvite, and cystine. Moreover, recent incidents such as the 2007 melamine pet food recall in North America [6] and the 2008 milk scandal in China [7] have also brought attention to the emergence of kidney stones caused by exposure to melamine (MEL) and cyanuric acid (CYA).

MEL at a certain dose of exposure has been proved to induce urolithiasis in both animal experiments and epidemiological studies [8]. MEL can form co-precipitates with uric acid or CYA (one of the main derivatives of MEL) [9,10,11]. Most importantly, although the acute toxicity of MEL and CYA alone is low, their combination dramatically increases the toxicity, as shown in animal tests producing nephrolithiasis of a 12–20-fold higher dose than MEL (or CYA) administrated alone [12,13].

It should be noted that human exposure to MEL and its derivatives in daily life is not limited to scenarios of illegal addition. We previously reported that environmental contamination, consumption of plant and animal foods, and exposure to cyromazine might be important sources of human MEL exposure [14]. Wu et al. [15,16] found that using melamine-made tableware in daily life increases MEL outputs, especially under high temperatures and high acidity conditions. Our recent population research indicated that MEL, CYA, and AMD were detected in >96% of urine samples [17]. Other studies have also shown that MEL can be discovered in most urine samples from the general population, indicating that it may be widely prevalent in the human body [18,19,20]. CYA is often used as an industrial raw material for bleach, fungicides, and herbicides. Human exposure to CYA primarily occurs through dichloroisocyanurate or trichloroisocyanurate, both of which serve as sources of active chlorine for disinfection purposes and can rapidly decompose into CYA when used in water [7,16,21]. Due to its high water solubility in environmental media, CYA exposure levels are usually 2–3 times higher than MEL [22,23]. These high levels of MEL and CYA have raised concerns about their co-exposure and potential health effects.

In the 2008 milk scandal in China, it was reported that MEL-associated kidney stones present with relatively mild clinical symptoms and have accessible treatment options [24]. The majority of affected children can be cured through simple medication, while a minority may require surgical interventions [25]. Current modalities for treating kidney stones include both surgical interventions and medication-based therapies [26]. Thiazide diuretics and potassium citrate are the primary medications utilized in the literature, but thiazide diuretics may cause adverse effects such as hypokalemia and intracellular acidosis, while potassium citrate may result in gastrointestinal discomfort, abdominal pain, and diarrhea [27]. It should be stated that, generally, only stones with mild symptoms can be treated medically. The reported surgical interventions generally include extracorporeal shockwave lithotripsy, percutaneous lithotripsy, and transurethral lithotripsy. However, surgical treatments are costly and may lead to acute kidney injury and high recurrence rates [28,29].

Recent research has demonstrated that tea and its specific active compounds possess mitigating or antagonistic properties against the toxicity of MEL. Salem RR [30] found that green tea extract (GTE) exhibited an antagonistic effect in response to MEL-induced liver toxicity. Compared with a group intoxicated with MEL, the group treated with a combination of MEL and GTE showed significant amelioration in histopathological changes induced by MEL in rat liver tissue. In another study, Li et al. [1] discovered that the formation of MEL–CYA complex crystals in a conditioned culture medium was pH-dependent. Additionally, it was found that catechin, an active compound present in tea, reduced the total number of crystals formed. In an in vivo investigation [1], the author observed that simultaneous oral administration of catechin via gavage effectively mitigated the renal crystal formation and toxicity induced by the MEL–CYA complex in Sprague–Dawley (SD) rats. This effect was attributed to the inhibition of reactive oxygen species (ROS) levels, cellular apoptosis, and signaling pathways involving phosphorylated p38 (p-p38) and osteopontin (OPN).

The aforementioned studies suggest that tea consumption or intake of tea-related extracts, such as catechin, holds potential for the prevention and treatment of MEL-associated kidney stones. However, limited evidence exists in the literature regarding this area (mainly by Li et al. [1]). Therefore, this study aimed to evaluate the therapeutic potential of catechin in treating nephrolithiasis induced by co-exposure to MEL and CYA in a rat model.

## 2. Materials and Methods

### 2.1. Experimental Animals

Male SD rats (n = 38) with an age of 6 weeks were purchased from Sippr-BK laboratory (Shanghai, China). These animals were housed in clean plastic cages containing wood shavings for bedding at 20~25 °C under a 12 h light/dark cycle and were fed a standard pelleted diet (Sippr-BK laboratory, Shanghai, China) and water ad libitum. The animals were acclimated for 7 days, at which point they were uniquely identified using ear tags. All animals received humane treatment in accordance with the guidelines approved by the Fudan University Animal Care and Use Committee (approval no. 2021JSSPH-010).

### 2.2. Experimental Design

**Figure 1:**
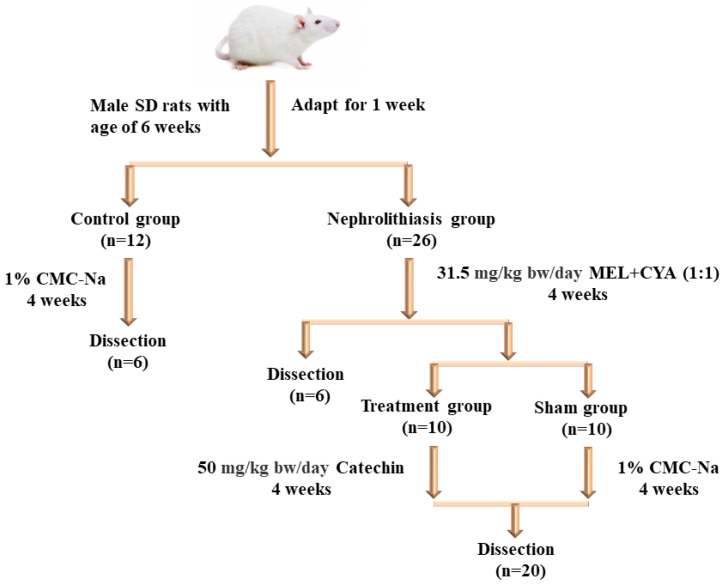
Experimental protocol for inducing and treating nephrolithiasis in SD rats.

The experimental procedure is illustrated in Figure 1. Rats were randomly divided into two groups: a nephrolithiasis group (n = 26) and a control group (n = 12). The nephrolithiasis group received melamine (31.5 mg/kg bw/day, by gavage) and then cyanuric acid (31.5 mg/kg bw/day, by gavage) 45 min later, which is approximately half of the no observed adverse effect level (NOAEL) value for MEL, as suggested by a 13-week gavage test of F344/N rats from the National Toxicology Program. This resulted in the formation of MEL + CYA crystals in their kidneys. Meanwhile, the control group was treated with a vehicle containing 1% carboxymethylcellulose sodium (CMC-Na). After a 28-day induction period, six rats from each group were selected for dissection, to confirm the successful induction of the kidney stone model. The remaining rats in the nephrolithiasis group were randomly assigned to two groups: (1) rats administered 1% CMC-Na vehicle (sham group; n = 10), and (2) rats administered 50 mg/kg bw/day catechin (treatment group; n = 10). Treatment was given for 28 consecutive days via gavage at a total volume of 1 mL/100 g·bw.

### 2.3. Test Material

Dosing solutions (*w*/*v*) of melamine (MEL; 99% pure; Sigma-Aldrich, St. Louis, MO, USA), cyanuric acid (CYA; 98% pure; Sigma-Aldrich, St. Louis, MO, USA), and catechin (98% pure; Aladdin, Shanghai, China) were prepared in 1% carboxymethylcellulose (CMC-Na; Aladdin, Shanghai, China).

### 2.4. Clinical Observation

The animals were subjected to daily clinical observation during gavage administration, with a focus on monitoring changes in skin, hair, eyes, and mucous membranes. Additionally, the somatic activity and mental state of the animals were observed, along with any signs of salivation, convulsions, diarrhea, or hematuria.

### 2.5. Tissue Collection

Following a 28-day induction period, six rats from the nephrolithiasis group and six from the controls were randomly selected for analysis. The animals were humanely euthanized through an overdose of 10% chloral hydrate. Subsequently, the kidneys were excised and evaluated for morphological alterations, with the weight recorded. Half of the kidneys were preserved in 10% formalin for histopathological analysis, while the other half were stored at −80 °C for subsequent wet mount analysis [31,32].

After administering catechin in model rats of nephrolithiasis, five rats from both the treatment and sham groups were randomly selected for a similarity analysis. The procedure for these animals followed the same protocols as outlined above.

### 2.6. Serum Chemistry

Blood was taken from the heart following anesthesia in the nephrolithiasis group and control group of rats. For the treatment period rats, orbital blood collection was conducted weekly following brief anesthesia with ether. Blood samples were centrifuged at 3500 RPM for 10 min, to separate the plasma and blood cells, then stored frozen at −20 °C for subsequent analysis of renal dysfunction markers, including serum creatinine (SCr), blood urea nitrogen (BUN), N-acetyl-β-D-glucosaminidase (NAG), β2-microglobulin (β2-MG), Cystatin C (CysC), kidney injury molecule 1 (KIM-1), and neutrophil gelatinase-associated lipocalin (NGAL).

### 2.7. Urine Collection

In rats with kidney stones during treatment, 24 h urine samples were obtained every three days utilizing metabolic cages. Urine samples were centrifuged at 3000 RPM for 10 min and subsequently stored at −20 °C for the analysis of renal dysfunction markers, including creatinine (Cr), NAG, β2-MG, CysC, KIM-1, and NGAL.

### 2.8. Histopathology

Kidneys were fixed in 10% formalin for 24 h, then sections were embedded in paraffin and stained with hematoxylin-eosin (HE) and Masson, and sections were viewed using a light microscope.

### 2.9. Macroscopic Evaluation of Renal Tissue (Wet Mount Preparation)

Frozen kidney tissues were used to generate wet mount sections, which were sectioned using a frozen sectioning machine. The sections were placed on slides, and phosphate buffer solution (PBS) buffer was added to facilitate the observation of any golden spherical crystals under a light microscope. The slides were then covered with coverslips, to ensure proper preservation of the tissue samples. Serial images were taken along the renal cortex under the microscope, and the crystals in the field of view were ranked from 0 to V, as follows: (0) none seen; (I) extremely few (1 or 2 in an entire area); (II) few with scattered distribution; (III) moderate numbers seen throughout section; (IV) large numbers seen immediately; and (V) extensive numbers obliterating the regular tissue architecture.

### 2.10. Statistical Analysis

Data are presented as the mean ± standard deviation (SD). Relative kidney weight (%) was evaluated using the following formula: relative kidney weight (%) = [kidney weight (g)/final body weight (g)] × 100 [1]. While evaluating the data, descriptive statistical methods and the distribution of the data were evaluated with a Shapiro–Wilk Test. Levene’s test was employed to examine the chi-square variance. According to the characteristics of the data, either an independent two-sample t-test or Mann–Whitney U-test was employed for analyzing the differences between the groups. Significance was evaluated at *p* < 0.05 level.

## 3. Results

### 3.1. Induction of Nephrolithiasis in Rats

**Figure 2:**
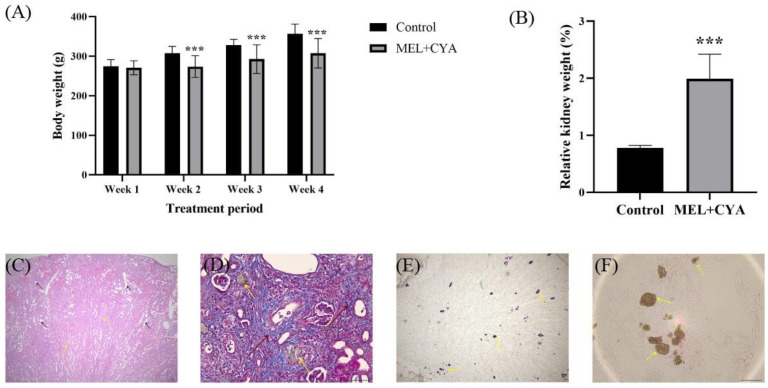
Anatomical data and pathological diagrams of the control group and nephrolithiasis group: (**A**) Body weight of rats in the control and nephrolithiasis group; (**B**) Relative kidney weight (%) of rats in the control and nephrolithiasis group; (**C**) HE section of rat kidney from the nephrolithiasis group; (**D**) Masson section of rat kidney from the nephrolithiasis group; (**E**,**F**) Kidney wet mount fresh tissue sections from the nephrolithiasis group. Black arrow: renal tubular dilatation; peach arrow: interstitial fibrosis; yellow arrow: MEL + CYA crystals. *** *p* < 0.001 indicates significant difference from control group (*p* < 0.05). Bar = 50 μm.

After three days of induction, about 1/6 rats in the nephrolithiasis group showed hematuria. Prolonged exposure to MEL + CYA resulted in gradual development of alopecia in the neck, legs, and abdomen of treated rats, with the affected areas becoming increasingly widespread. No abnormalities were observed in the control group rats during the induction period. Compared to the control group, rats in the nephrolithiasis group exhibited a significantly lower body weight starting from the second week of induction. Additionally, the post-autopsy analysis revealed a significant increase in the relative kidney weight (%) (Figure 2A,B). Moreover, histopathological examination of the kidney samples demonstrated severe damage, characterized by extensive tubular dilatation, focal interstitial fibrosis, and crystal deposition (Figure 2C–F).

### 3.2. Effect of Catechin Treatment on Body Weight and Renal Index

**Figure 3:**
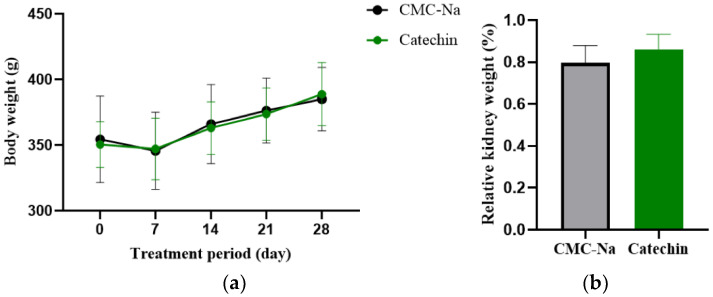
Body weight and relative kidney weight of rats during treatment: (**a**) Body weight after administration of MEL + CYA; (**b**) Relative kidney weight (%) in the CMC-Na (sham group) and catechin (treatment group).

The treatment and sham groups did not exhibit any significant variations in body weight and renal indices (Figure 3). Rats in the treatment group, which experienced alopecia during the induction phase, demonstrated a regrowth of dense fur on their heads and abdomens during the treatment period.

### 3.3. Effect of Catechin Treatment on Crystal Deposition in the Kidneys

**Figure 4:**
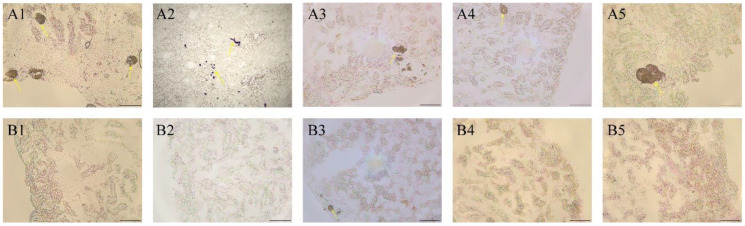
Kidney wet mount fresh tissue sections from rats treated with CMC-Na (sham group) or given with oral catechin at 50 mg/kg bw/day (treatment group). (**A1**–**A5**) represent the wet mount section plots of five rats in the sham group, respectively; (**B1**–**B5**) represent the wet mount section of five rats in the treatment group, respectively. Yellow arrow: MEL + CYA crystals; Bar = 50 μm.

Sequential mapping was conducted along the renal cortex to observe changes in the quantity and density of MEL + CYA crystals. The results indicated a significant reduction in both crystal density and quantity in the treatment group compared to the control group. Table 1 summarizes the crystal intensity and presence for all tested animals. Crystal deposition was observed in all rats from the control group 28 days after staining, with a large number of crystals visible under microscopy. In contrast, only one rat from the treatment group exhibited a few crystals (Figure 4). These findings suggest that catechin had a positive effect on MEL + CYA crystal degradation and reduced their quantity.

### 3.4. Effect of Catechin Treatment on Kidney Histopathology

**Figure 5:**
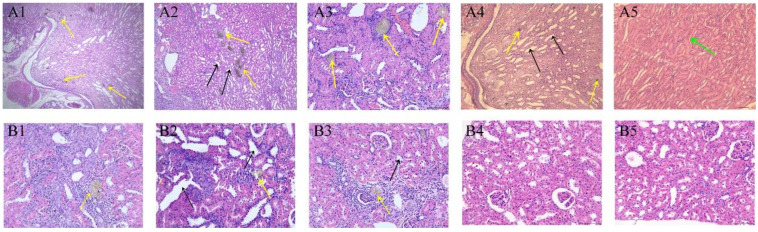
HE sections of kidneys of rats treated with CMC-Na (sham group) or given oral catechin at 50 mg/kg bw/day (treatment group). (**A1**–**A5**) represent the HE sections of different rats in the sham group; (**B1**–**B5**) represent the HE sections of different rats in the treatment group. Black arrow: renal tubular dilatation; Green arrow: interstitial congestion; Yellow arrow: MEL + CYA crystals. Bar = 50 μm.

**Figure 6:**
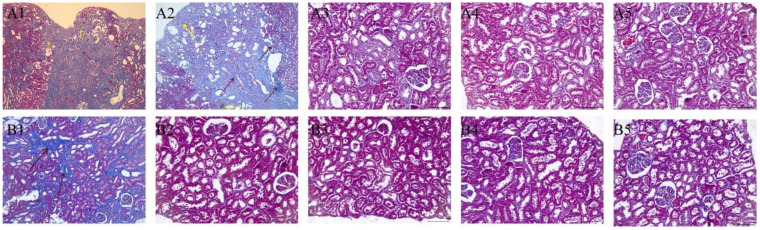
Masson sections of kidneys of rats treated with CMC-Na (sham group) or given oral catechin at 50 mg/kg bw/day (treatment group). (**A1**–**A5**) represent the Masson sections of different rats in the sham group; (**B1**–**B5**) represent the Masson sections of different rats in the treatment group. Peach arrow: interstitial fibrosis; Yellow arrow: MEL + CYA crystals. Bar = 50 μm.

In the sham group, significant histopathological damage was observed, including varying degrees of tubular dilatation and crystal deposition. In the sham group, significant dilation of the renal papilla-collecting duct region was observed, accompanied by crystal deposition within the lumen (Figure 5(A1,A4)). Furthermore, multiple instances of renal parenchymal fibrosis (Figure 6(A1,A2)) and focal interstitial congestion (Figure 5(A5)) were identified. In contrast, no abnormalities were observed in two rats in the treatment group, while the remaining rats exhibited only slight tubular dilation and occasional crystal deposits (Figure 5(B1–B3)), as well as focal interstitial fibrosis (Figure 6(B1)). These results demonstrate the protective effect of catechin against histopathological damage caused by MEL + CYA crystal formation and suggest its potential as a therapeutic intervention.

### 3.5. Effect of Catechin Treatment on Renal Function Parameters

**Figure 7:**
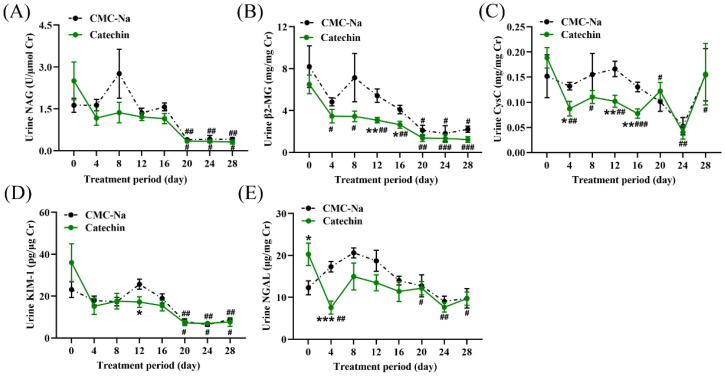
Urinary biochemical levels in the sham and treatment groups during the treatment period. (**A**–**E**) represent the variations in urinary biochemical levels of NAG, β2-MG, CysC, KIM-1, and NGAL during the treatment period for both the sham and treatment groups. * *p* < 0.05, ** *p* < 0.01, *** *p* < 0.001 indicate a significant difference between the sham group and the treatment group (*p* < 0.05). ^#^ *p* < 0.05, ^##^ *p* < 0.01, ^###^ *p* < 0.001 indicate a significant difference from respective baseline (*p* < 0.05).

The effect of the catechin on the urinary renal function parameters was assessed (Figure 7). During the treatment period, there was a gradual decrease observed in all urinary renal function parameters for both the treatment and sham groups. On day 4, urinary CysC levels were significantly lower in the treatment group, while on days 12 and 16 urinary β2-MG and CysC levels were significantly lower compared to the sham group. Additionally, the baseline urinary NGAL levels were significantly higher in the treatment group compared to those in the sham group. However, after three days of catechin treatment, there was a significant decrease in urinary NGAL levels in the treated group compared to its baseline, as well as being significantly lower than that observed in the sham group. It was not until day 20 that significant decreases were observed in the urinary NAG, β2-MG, and KIM-1 levels within the sham group when compared to their respective baseline values. The urinary renal function parameters of the treatment group were significantly reduced at the end of the treatment period compared to their starting point.

**Figure 8:**
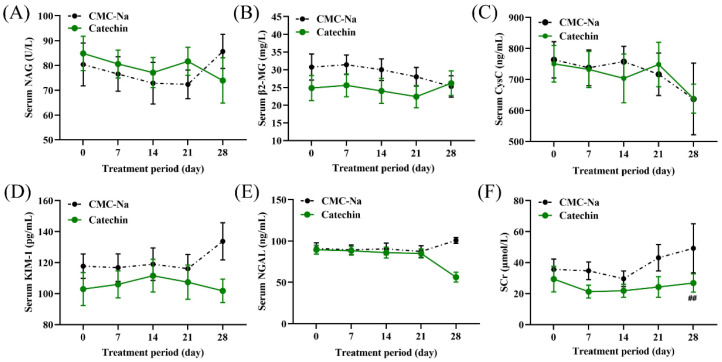
Serum biochemical levels in the sham and treatment groups during the treatment period. (**A**–**E**) represent the variations in serum biochemical levels of NAG, β2-MG, CysC, KIM-1, NGAL and SCr during the treatment period for both the sham and treatment groups. ^##^ *p* < 0.01 indicates a significant difference from respective baseline (*p* < 0.05).

Serum creatinine in the fourth week was significantly reduced in the treatment group compared with the sham group (Figure 8). No significant changes were found in other renal function parameters of serum biochemistry.

## 4. Discussion

In this study, we found that catechin presented a therapeutic effect in reversing crystal deposition, histopathological damage, and renal dysfunction. This is the first in vivo animal study, to our knowledge, to demonstrate a therapeutic effect in treating nephrolithiasis. Previous research conducted by Li et al. [1] also reported that catechin consistently and significantly reduced levels of renal crystals and nephrotoxicity when co-administered with a MEL–CYA mixture in both in vitro and in vivo studies. Our study differed from Li et al.’s in vivo study in two aspects of the methodology: (1) we separately administered MEL and CYA to SD rats, while they used a pre-prepared MEL–CYA solution that could form a melamine–cyanurate complex in vitro; (2) we administered catechin to rats with nephrolithiasis pre-induced by MEL + CYA co-exposure, while they co-exposed catechin with melamine–cyanurate complex to rats.

The therapeutic efficacy of catechin in the treatment of nephrolithiasis/urolithiasis induced by co-exposure to MEL and CYA can be attributed to its potent antioxidant properties [33,34,35], which facilitate stone ablation through two pathways:

The first pathway of stone ablation that may be modulated by the antioxidant properties of catechin is the alleviation of oxidative stress (OS) in the kidney. Studies have shown that long-term exposure to MEL + CYA can induce OS in the kidney, leading to damage, apoptosis, and inflammation of renal tubular epithelial cells, which may contribute to melamine-related kidney stone formation [36]. Li et al. [37] reported that the selective NADPH oxidase inhibitor, apocynin, can prevent MEL-induced kidney stone formation by reducing OS. In the same year, Li et al. [1] also confirmed that catechin can normalize MEL + CYA-induced ROS levels and decrease expression of OPN and p-p38 associated with crystal formation and retention [38,39]. In addition to its efficacy against MEL-associated kidney stones, catechin has demonstrated potential in inhibiting the formation of calcium oxalate crystals within the renal system, potentially through its antioxidative properties, which protect against the oxidative damage inflicted upon the lipid peroxidation and apical epithelium of the renal tubular membrane surface [40,41].

Another pathway of stone ablation that may be modulated by the antioxidant properties of catechin involves reducing physical damage to renal tissue and thereby decreasing the process of crystal attachment or anchoring at the site of injury. When the renal tubular rim is compromised, crystals can adhere to damaged tubular cell surfaces and cannot be excreted in urine [33]. The preliminary investigation conducted by Randall revealed a strong correlation between crystal adhesion to the renal papillae and tissue damage [42]. Kanlaya et al. [43] discovered that catechin inhibits the binding of calcium oxalate monohydrate crystals to renal tubular cells in vitro. Our observations following catechin treatment revealed a certain degree of tissue recovery, including renal tubular dilation and reduction in renal fibrosis in rats, indicating that catechin could enhance therapeutic efficacy against kidney stones by repairing renal tissue damage and reducing crystal adhesion sites. These findings are consistent with previous studies [33,44,45]. Moreover, the treatment group exhibited a significant reduction in renal function parameters such as urinary NAG and urinary β2-MG compared to post-poisoning levels, indicating the mitigating effect of catechin on kidney toxicity [33].

Furthermore, previous studies demonstrated that both catechin and citrate exhibit similar inhibitory mechanisms against MEL–CYA stone formation [1,46]: (1) in vitro experiments revealed that the addition of sodium citrate and catechin effectively suppressed MEL–CYA crystal formation when the pH was below 7; (2) animal experiments indicated that both catechin and sodium citrate significantly inhibited melamine–cyanuric acid-induced OPN expression, which is closely associated with crystal formation and retention [47]. Considering the widespread clinical use of citrate for kidney stone treatment, due to its ability to alkalinize urine and reduce urine saturation [48], it is worth exploring whether catechin can also alleviate kidney stones by modulating urinary alkalinity and mitigating the physical as well as chemical risk factors involved in stone formation.

Another point that needs to be discussed is the dose of 50 mg/kg bw/day used in our study. If the conversion of this dose to a human equivalent is calculated using the Meeh–Rubner equation, based on the relative ratio of body surface area per body weight and the K value of 6.3 for rats to humans, then an individual weighing 60 kg would require approximately 480 mg of catechin daily [49,50]. According to a previous study [51], it has been determined that a standard cup of green tea prepared using 2.5 g of tea leaves (250 mL) typically contains approximately 240 to 320 mg of catechin. Considering an individual weighing 60 kg, the consumption of two cups of green tea per day could be deemed sufficient for achieving the above dosage of 480 mg/day. That means the human behavior of drinking green tea may have the therapeutic capacity for treating nephrolithiasis/urolithiasis induced by co-exposure to MEL and CYA.

Our study has some major limitations that need to be addressed. First, the causal relationship obtained from animal experiments is limited. Second, we did not explore research on the reason of why catechin presented a capability for alleviating kidney stones, especially the molecular mechanism for oxidative stress, fibrosis, etc. Third, only one dose of catechin was used in our study.

## 5. Conclusions

The present study presented data of pioneering evidence on catechin’s capacity for treating nephrolithiasis induced by co-exposure to MEL and CYA. Considering that tea is a significant source of catechin and tea consumption is a prevalent global practice, our study may present a good strategy for the prevention of MEL-associated nephrolithiasis and urolithiasis. Future research, including both animal experiments and epidemiologic studies, is warranted for investigating the role and mechanism of tea consumption in treating MEL-associated urolithiasis.

## Figures and Tables

**Table 1:** Intensity and presence of MEL + CYA crystals in the sham group and treatment group rats after 28 days.

Group	Crystal Intensity *	Total Number of Animals with Crystals
Sham (CMC-Na)	III, IV, II, II, I	5 of 5
Treatment (Catechin)	0, 0, I, 0, 0	1 of 5

* The methodology of ranking crystal density was described in the methods section (2.9 Macroscopic evaluation of renal tissue).

## Data Availability

The datasets of this study are available from the corresponding author on reasonable request.
